# Supplementary material for: Is teenage parenthood associated with early use of disability pension? Evidence from a longitudinal study
Source: PLoS One. 2023 Jun 14;18(6):e0287265. doi: 10.1371/journal.pone.0287265 (PMC10266663; doi:10.1371/journal.pone.0287265)
Supplement: Attachment — Submitted filename: REVIEW_Moon_2022-0905_final.docx [file pone.0287265.s001.docx]

# REVIEW_MOON

# This is an interesting study conducted on early application and use of disability pensions (DP) for teenage parents. Below, I address some of the problems/questions that appeared when I read the paper.

# Introduction

In the introduction, the study reported that the proportion of disability pensions (DP) in several European countries is increasing, and according to the WHO, one in seven people (14%) experience mental illness, accounting for 13% of the global burden caused by disease within this age group. It was said that this mental health had a negative effect, and the risk of higher health deterioration and socioeconomic consequences was high in teenage parents.

This high mental health problem can be identified as a strong predictor for using disability pension (DP) and hypothesized that becoming a teenage parent increases the risk of receiving disability pension (DP) in adulthood, and for the purpose of the study, it was examined whether there was a difference in the use of early disability pension (DP) between parents.

However, based on previous studies and introductions, it can be confirmed that there are already many mental health risks in adolescent parents, and as a result, it can be predicted that the probability of receiving a disability pension (DP) will be high.

Therefore, it is not well felt whether this content is an important explanatory element for research. If the research problem and hypothesis can be sufficiently confirmed based on the existing research, it will be necessary to derive and prove a more detailed research hypothesis.

I propose to mention the importance of research hypotheses in the discussion. In other words, why should readers know the finding of the hypothesis established by the researcher?

# Results

# The results reported in this study used analysis techniques through Kaplan-Meier curves and Cox provincial regression, but it was shown as an occurrence rate over simple time, and statistical significance verification of the results was not confirmed. There will be a need for solid information about the scientific validity of this report.

# Discussion

This study reported that through discussion, the probability of teenage parents using disability pension (DP) and important results that teenage parents later adversely affect mental health were confirmed as implications.

If other research results or cohort comparison data that can support these implications were explained more abundantly, the validity of the results according to the research purpose and research hypothesis could be improved. However, since this research data is simple technical information, it is questionable whether it is information that can generalize the research results.

# Conclusions

In conclusion, teenage parents are likely to start using the disability pension (DP) at the age of 20-42. However, it does not explain why teenage parents apply for a disability pension (DP) between the ages of 20-42.

In addition, it is based on Swedish cohort research data, and since Sweden's support policy is richer than other countries, the conclusion that other countries will have relatively larger problems is insufficient and it will need to be verified through comparison between countries in the future.
